# Stevens-Johnson syndrome/toxic epidermal necrolysis induced by sintilimab in a patient with advanced non-small cell lung cancer: A case report

**DOI:** 10.1097/MD.0000000000047805

**Published:** 2026-02-20

**Authors:** Yanli Zhao, Jianchun Duan, Guoping Tong, Wenzhong Su, Hui Wang, Fangfang Wu, Chen Liang, Lingling Zhao, Tong Zhou, Jinfang Zhai

**Affiliations:** a Department of Respiratory Medicine, Shanxi Cancer Hospital, Shanxi, China; b University of Bristol, Bristol, United Kingdom; c Kindstar Global Precision Medicine Institute, Wuhan, China; d Wuhan Kindstar Zhenyuan Medical Laboratory Co. Ltd, Wuhan, China.

**Keywords:** immune-related adverse events, non-small cell lung cancer, sintilimab, Stevens-Johnson syndrome, toxic epidermal necrolysis

## Abstract

**Rationale:**

Sintilimab, an immune checkpoint inhibitor, enhances T-cell responses, leading to robust antitumor activity, and is approved for the treatment of lung cancer. While immune checkpoint inhibitors offer substantial clinical benefits, they are often associated with immune-related adverse events, particularly cutaneous toxicities. These skin reactions typically manifest as mild maculopapular rashes; however, more severe manifestations, such as Stevens–Johnson syndrome (SJS) and toxic epidermal necrolysis (TEN), can occur. Both SJS and TEN are life-threatening conditions with high mortality rates.

**Patient concerns:**

The patient in this case is a 68-year-old male diagnosed with stage IVA non-small cell lung cancer, who has no detectable oncogenic mutations. He was treated with sintilimab (200 mg), pemetrexed disodium (500 mg/m^2^), and cisplatin (75 mg/m^2^). Following the third cycle of therapy, he developed widespread skin blisters, localized necrosis, and severe pain on the second day.

**Diagnoses:**

The patient’s symptoms – widespread skin blisters, localized necrosis, and severe pain – were indicative of SJS and TEN, which are immune-related cutaneous toxicities triggered by immunotherapy.

**Interventions:**

Immediate interventions were initiated, which included systemic corticosteroids, anti-inflammatory treatments, fluid replacement, and appropriate skin care measures.

**Outcomes:**

These interventions led to significant improvement in the immunotherapy-related dermatologic toxicity experienced by the patient.

**Lessons:**

This case underscores the importance for clinicians to remain vigilant regarding severe cutaneous toxicities such as SJS and TEN in patients undergoing sintilimab-based therapy. Prompt recognition and intervention are essential to mitigate the risks associated with these potentially life-threatening conditions.

## 1. Introduction

Immune checkpoint inhibitors (ICIs) have revolutionized the treatment of advanced and metastatic cancers, particularly that refractory to conventional therapeutic modalities. However, their inherent immunomodulatory properties predispose patients to immune-related adverse events (irAEs), among which severe cutaneous toxicities represent a clinically significant subset. By activating T cells systemically across multiple tissues compartments, ICIs can trigger aberrant immune overactivation – a key pathogenic driver of irAEs that most commonly affect the skin, colon, liver, lungs, endocrine organs, and joints, while potentially affecting nearly all organ system.^[1,2]^ Recent bibliometric analyses underscore the escalating global attention to immunotherapy-related complications in oncology practice, emphasizing the clinical relevance of rare yet life-threatening irAEs despite their low overall incidence.^[3,4]^ Furthermore, accumulating evidence reveals that metabolic reprogramming in lung cancer – defined by dysregulated glycolysis, glutaminolysis, and nutrient competition within the tumor microenvironment – modulates both ICIS therapeutic efficacy and susceptibility to immune-related toxicities, thereby offering critical mechanistic insights into the pathogenesis of these adverse events.^[5]^

Despite the rapidly expanding clinical utilization of ICIs, a notable gap remains in the literature concerning their dermatologic toxicities, particularly regarding severe cutaneous adverse reactions, including Stevens-Johnson syndrome (SJS) and toxic epidermal necrolysis (TEN). These life-threatening mucocutaneous disorders are of special concern in immunocompromised cancer patients, given their substantial morbidity and mortality. Limited data on ICI-induced SJS/TEN, and the absence of well-defined clinical criteria to differentiate them from conventional drug-induced variants presents substantial diagnostic and therapeutic dilemmas. Thus, additional research is essential to improve the recognition and management of these severe immune-related dermatologic toxicities, as well as to gain a better understanding of their implications for patient outcomes. Herein, we present a case of severe immune-related SJS/TEN in a patient with stage IVA non-small cell lung cancer (NSCLC) treated with sintilimab in combined with pemetrexed disodium and cisplatin.

## 2. Case presentation

A 68-year-old male presented to Shanxi Cancer hospital on April 12, 2023, with a 4-month history of cough, sputum production, and dyspnea. Initially, he experienced a nonproductive, irritating cough that developed into production cough with white, sticky sputum, and exertional dyspnea. The patient reported no identifiable triggers and denied fever, hemoptysis or chest pain. Upon physical examination, his skin and mucous membranes were unremarkable, without signs of erythema, swelling, or rash. No palpable lymph nodes were detected in the bilateral cervical, supraclavicular, or axillary regions. The chest was symmetrical, with equal vocal fremitus bilaterally. Auscultation revealed clear breath sounds without adventitious sounds such as rales. Table 1 depicts the timeline of the patient.

**Table 1:** Timeline of a case with lung adenocarcinoma complicated by SJS/TEN.

Data	Symptoms	Interventions	Outcomes
April 13, 2023	Cough, sputum, and dyspnea	Chest CT, MRI, bone scan, abdominal US, and pathology	Left lower lobe adenocarcinoma (metastatic, cT2N3M1a, and IVA)
July 10, 2023	/	Treatment: 2 cycles of immunochemotherapy	Stable disease
July 12, 2023	Widespread skin blisters, necrosis, and severe pain	Systemic steroids, antibiotics, fluid replacement, and wound care	SJS/TEN (Grade 4)
July 20, 2023	Extensive blisters, severe pain, and fatigue	Blisters aspiration, 0.9% normal saline irrigation, pressure ulcer prevention and wound care	/
July 31, 2023	Blisters resolving, desquamation, and local bleeding/exudation	Continued wound irrigation care	Condition improved
August 9, 2023	Pain reduced, bleeding/exudation decreased, and pink granulation	Continued wound care	Discharge
September 10, 2023	Wound healed and mild cutaneous pigmentation	/	Treatment satisfactory

MRI = magnetic resonance imaging.

On April 13, 2023, a chest CT revealed irregular nodular opacities in the left lower lung lobe, bilateral supraclavicular and mediastinal lymphadenopathy, and left-sided pleural effusion. Further imaging, including cranial magnetic resonance imaging, whole-body bone scintigraphy, and abdominal ultrasound, showed no evidences of distant metastatic disease. Bronchoscopy indicated mild bronchial mucosal swelling of the left bronchus. Subsequent CT imaging demonstrated an irregular, spiculated lesion with heterogeneous density in the left lower lobe. Pathological examination of bronchoalveolar lavage fluid revealed a small population of deeply stained heterogeneous cells with large nuclei, suggestive of malignancy. Closed thoracic drainage was performed for pleural effusion management, and adenocarcinoma cells were identified in the pleural fluid across 3 separate cytological evaluations. During hospitalization, the patient remained afebrile with normal white blood cell counts. Ultimately, a diagnosis of left lower lobe adenocarcinoma with mediastinal and bilateral supraclavicular lymph node metastasis, as well as left pleural metastasis (stage cT2N3M1a, IVA) was confirmed. The patient had an Eastern Cooperative Oncology Group performance status (PS) of 1. Next-generation sequencing was performed, but no driver gene mutations were identified.

On July 10, 2023, after completing 2 cycles of combination immunochemotherapy consisting of Sintilimab (200 mg), pemetrexed disodium (500 mg/m^2^), and cisplatin (75 mg/m^2^), the patient’s disease was assessed as stable, which prompted the initiation of the third cycle.

On July 12, 2023, the patient developed widespread skin blisters with focal necrosis and severe pain, consistent with a diagnosis of the Common Terminology Criteria for Adverse Events (CTCAE) grade 4 immunotherapy-induced SJS/TEN. Prompt interventions encompassed systemic methylprednisolone (2 mg/kg), cefoperazone sodium-sulbactam sodium (3 g q12h), fluid replacement, and intensive cutaneous care. Although exposed to 3 antineoplastic agents, sintilimab is identified as the causative agent: Cytotoxic chemotherapeutics (pemetrexed disodium and cisplatin) typically elicit acute cutaneous reactions following initial administration, yet the patient exhibited no such manifestations after 2 cycles.^[6]^ ICI-related cutaneous toxicities commonly manifest 1 to 6 months post-initiation, aligning with the patient’s clinical course.^[7]^ The patient achieved complete resolution with systemic steroids, whereas chemotherapeutic-induced tissue damage is often irreversible with sequelae (e.g., scarring), which were not observed here.^[8,9]^ Collectively, these observations strongly implicate sintilimab as the etiologic agent.

**Figure 1.**
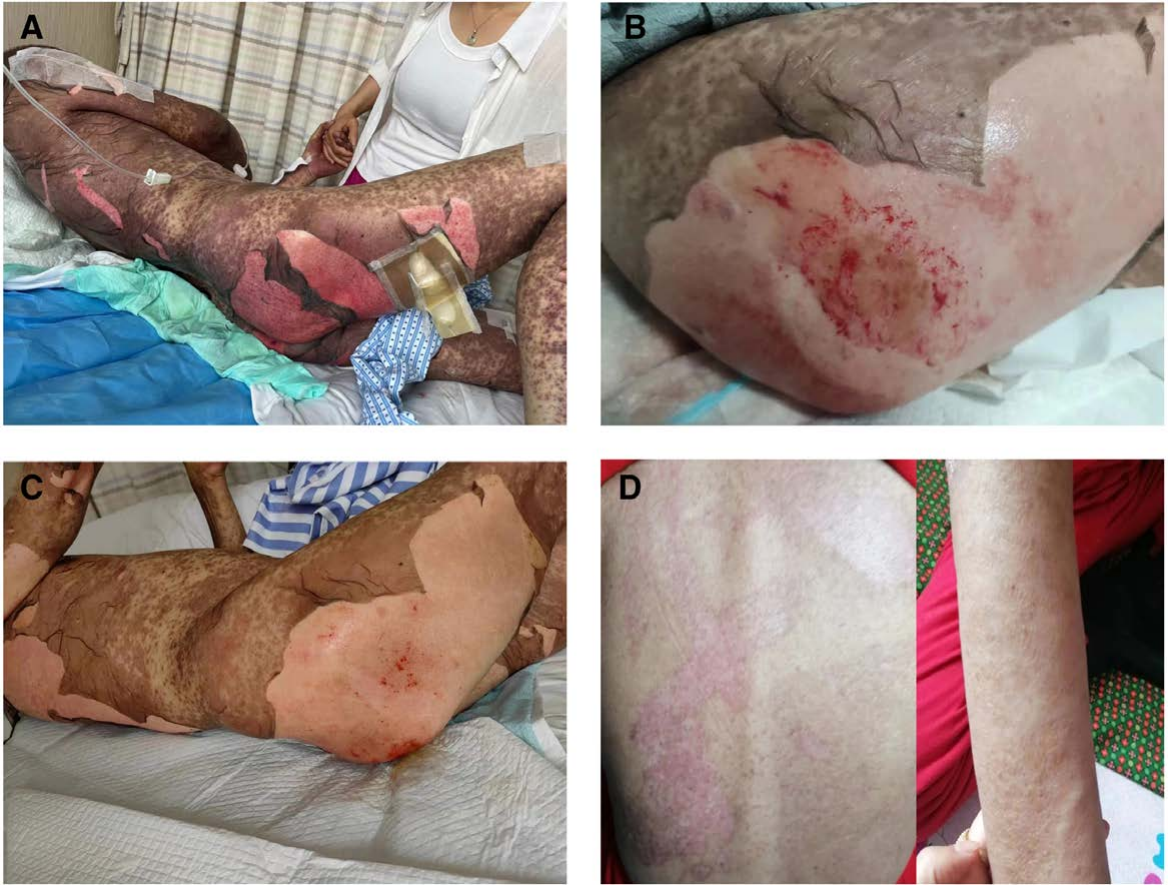
The figure depicts the changes in the skin condition of the patient before and after treatment. (A) Extensive coalescent blistering, accompanied by significant pain and fatigue, consistent with sintilimab-induced SJS/TEN. (B) Blistering subsided, with residual widespread desquamation, localized bleeding, exudation, and manageable persistent pain. (C) Significant improvement, including reduced pain, less bleeding and exudate, and formation of healthy pink granulation tissue. (D) Complete wound healing with residual cutaneous pigmentation; no symptom recurrence. SJS = Stevens-Johnson syndrome, TEN = toxic epidermal necrolysis.

On July 20, 2023, the patient presented with extensive blistering that gradually coalesced, accompanied by significant pain and fatigue (Fig. 1A). Key interventions blisters disinfection and aspiration, followed by irrigation with 0.9% saline. The wounds were dressed with oil gauze and overlaid with dry gauze. To prevent pressure ulcers, the patient was placed on an air-cushion bed and regularly repositioned every 2 hours. Nonpressure areas were treated with oil gauze impregnated with human epidermal growth factor (hEGF) gel and silver sulfadiazine ointment, with dressing changes performed twice daily. Oral and urethral hygiene was maintained via 0.9% normal saline to prevent secondary infections.

On July 31, 2023, blistering began to subside, although the patient continued to experienced widespread desquamation, localized bleeding, and exudation (Fig. 1B). Despite pain persisted, it became more manageable. Management was continued with wound irrigation and dressing changes, utilizing oil gauze impregnated with hEGF gel and silver sulfadiazine ointment.

On August 9, 2023, significant improvements were observed, including reduced pain, decreased bleeding and exudate, and formation of healthy pink granulation tissue (Fig. 1C). Daily wound care and dressing changes were maintained to support ongoing healing; additionally, the patient was encouraged to engage in moderate physical activity to improve circulation and facilitate tissue recovery. The patient was eventually discharged after pain resolution and elimination of analgesics requirements.

On September 10, 2023, a follow-up examination indicated that the patient’s wounds had healed, with residual cutaneous pigmentation (Fig. 1D). The patient reported no symptom recurrence and expressed satisfaction with the treatment outcomes.

## 3. Discussion

SJS/TEN is a rare yet life-threatening mucocutaneous drug reaction, characterized by low incidence, rapid progression, and high mortality.^[10]^ As a developed programmed cell death protein 1 (PD-1) inhibitor in China, Sintilimab was first approved on December 24, 2018, for the treatment of relapsed or refractory classical Hodgkin lymphoma (r/r cHL). Its combination with chemotherapy has since shown robust antitumor efficacy in NSCLC, with objective response rates exceeding 50% in phase III trials.^[11]^ However, reports of sintilimab-induced SJS/TEN remain scarce. A systematic search of PubMed, Embase, and China National Knowledge Infrastructure up to June 2024 identified only 18 cases globally, with merely 5 originating from China. This scarcity of data underscores the value of our case, as it highlights a critical safety consideration in the real-world application of this widely prescribed immunotherapeutic agent.

In our patient, severe irAEs developed and progressed to CTCAE grade 4 SJS/TEN 66 days after initiating sintilimab-based combination immunochemotherapy. Existing evidence indicates that most immunotherapy-associated cutaneous toxicities occur within 1 to 6 months of treatment initiation, with approximately 80% of SJS/TEN cases presenting within the first 2 months.^[12,13]^ Our patient’s onset at day 66, while within this broader window, occurred later than most reported cases – an observation that led our team to hypothesize whether concurrent chemotherapy (pemetrexed disodium and cisplatin) might have modulated the timing of irAE development. Unlike the acute cutaneous reactions associated with pemetrexed or cisplatin (which typically emerge within 1–2 weeks of the first dose, often as mild maculopapular rashes), the delayed onset of SJS/TEN in our case is more closely with the immunological mechanism of PD-1 inhibitor-related toxicities: gradual dysregulation of T-cell homeostasis and subsequent hyperactivation of immune responses against cutaneous antigens. This distinction reinforces a key clinical insight from our experience: when managing patients on immunochemotherapy, clinicians must monitor not only for early chemotherapy-related reactions but also remain vigilant for delayed irAEs, even beyond the “typical” 2-month window.

The patient achieved significant clinical improvement with high-dose systemic corticosteroid (2 mg/kg methylprednisolone sodium succinate), followed by a gradual tapering regimen.^[14]^ While intravenous immunoglobulin (IVIG) and cyclosporine are recommended in some guidelines for severe SJS/TEN,^[15]^ we chose not to use these agents based on 2 key considerations. First, a 2018 systematic review^[16]^ found that IVIG provided no significant mortality benefit in PD-1 inhibitor-induced SJS/TEM compared with high-dose steroids alone, particularly in patients without evidence of multiorgan failure. Second, from a practical clinical perspective, IVIG is associated risk of volume overload and considerable cost burden. Instead, we incorporated topical recombinant hEGF gel, which stimulates the synthesis of DNA, RNA, and hydroxyproline to accelerate granulation tissue formation and promote the proliferation of wound epithelial cells. This approach resulted in shorter wound healing time in our patient, which likely reduced his risk of secondary infections. For wound care, we also utilized a combination of sulfadiazine and silver salts, which exhibit broad-spectrum antimicrobial activity against Gram-positive bacteria, Gram-negative bacteria, yeasts, and fungi – pathogens that are commonly responsible for fatal sepsis in patients with SJS/TEM. Additionally, silver salts exert an astringent effect that aids wound drying and promotes scab formation, thereby enhancing the healing process. To complement these interventions, we implemented rigorous oral and urethral hygiene with 0.9% normal saline and closely monitored fluid intake and output to maintain electrolyte and acid-base balance. These measures were particularly important given the patient’s high-dose steroid use, which posed a risk of immunosuppression and fluid retention. In our clinical practice, such “supportive care optimization” has proven just as critical as targeted anti-irAEs therapy for improving patient outcomes.

A final key consideration from our case is the decision to permanently discontinue ICIs therapy following CTCAE grade 4 SJS/TEM.^[17]^ While guidelines uniformly recommend permanent discontinuation for grade 4 irAEs, this decision presented a unique clinical dilemma: our patient had stage IVA NSCLC without actionable driver mutations, leaving ICIs therapy among his limited effective treatment options. Our multidisciplinary team discussed the feasibility of a potential future trial of low-dose ICIs with prophylactic steroids, but ultimately prioritized safety, given that SJS/TEN recurrence rates exceed 30% in patients who resume ICIs therapy, with even mild recurrences capable of rapidly progress to life-threatening severity. Instead, once the patient’s PS recovered to Eastern Cooperative Oncology Group PS 1, we selected a limited course of systemic chemotherapy (pemetrexed monotherapy), a choice that balanced disease control with the minimization of further toxicity. This experience highlights the need for more personalized strategies to ICIs rechallenge in patients with rare, severe irAEs, an area in which additional prospective studies are urgently required.

In conclusion, our case describes a rare late-onset CTCAE grade 4 SJS/TEN induced by sintilimab, successfully managed with high-dose steroids, targeted topical therapy, and meticulous supportive care – without the need for IVIG or cyclosporin –thus providing a practical therapeutic alternative for resource-limited settings or patients intolerant to conventional immunomodulators. As ICIs are increasingly utilized in oncology clinics across China, clinicians must maintain heightened vigilance for SJS/TEN, with key strategies encompassing pretreatment allergic history evaluation, biweekly skin assessments, and prompt interdisciplinary collaboration between oncologists and dermatologists at the first sign of severe rash. Future research should prioritize the identification of predictive biomarkers for ICI-induced SJS/TEN and assess the efficacy of novel immunomodulators agents in refractory cases to balance the remarkable efficacy of ICIs with optimal patient safety. While this case offers valuable clinical insights, we acknowledge its limitations: the single-case design limits generalizability, histopathological confirmation via skin biopsy was not performed (diagnosis was based on clinical manifestations and temporal association with sintilimab exposure), repeat diagnostic tests (e.g., lymphocyte transformation assay) or biomarker analyses were not conducted to corroborate causality, and the absence of long-term follow-up data precludes assessment of potential late sequelae or the safety of subsequent ICIs rechallenge. Despite these constraints, our experience underscores the critical need for vigilance against severe cutaneous adverse reactions during ICIs therapy and adds to the accumulating evidence on alternative treatment strategies for ICI-induced SJS/TEN.

## Acknowledgments

We thank the patient for her cooperation and support for academic communication.

## Author contributions

**Conceptualization:** Yanli Zhao, Jianchun Duan, Guoping Tong, Jinfang Zhai.

**Funding acquisition:** Yanli Zhao, Jianchun Duan, Jinfang Zhai.

**Investigation:** Wenzhong Su, Hui Wang, Fangfang Wu, Chen Liang.

**Methodology:** Yanli Zhao, Guoping Tong.

**Resources:** Jianchun Duan, Fangfang Wu, Chen Liang, Lingling Zhao, Tong Zhou, Jinfang Zhai.

**Supervision:** Jinfang Zhai.

**Writing – original draft:** Yanli Zhao, Guoping Tong.

**Writing – review & editing:** Jianchun Duan, Wenzhong Su, Hui Wang, Fangfang Wu, Chen Liang, Lingling Zhao, Tong Zhou, Jinfang Zhai.
